# Efficacy of Cardiopulmonary Resuscitation Using Automatic Compression—Defibrillation Apparatus: An Animal Study and A Manikin-Based Simulation Study

**DOI:** 10.3390/jcm12165333

**Published:** 2023-08-16

**Authors:** Woo Jin Jung, Young-Il Roh, Hyeonyoung Im, Yujin Lee, Dahye Im, Kyoung-Chul Cha, Sung Oh Hwang

**Affiliations:** 1 Department of Emergency Medicine, Yonsei University Wonju College of Medicine, Wonju 26426, Republic of Korea; wjjung21c@yonsei.ac.kr (W.J.J.); md.youngilroh@yonsei.ac.kr (Y.-I.R.); ihy@yonsei.ac.kr (H.I.); eujin2452@yonsei.ac.kr (Y.L.); 1mdahye@yonsei.ac.kr (D.I.); 2 Research Institute of Resuscitation Science, Yonsei University Wonju College of Medicine, Wonju 26426, Republic of Korea

**Keywords:** cardiopulmonary resuscitation, cardiac arrest, defibrillation, medical device, Hemodynamics

## Abstract

Background: Chest compression and defibrillation are essential components of cardiac arrest treatment. Mechanical chest compression devices (MCCD) and automated external defibrillators (AED) are used separately in clinical practice. We developed an automated compression–defibrillation apparatus (ACDA) that performs mechanical chest compression and automated defibrillation. We investigated the performance of cardiopulmonary resuscitation (CPR) with automatic CPR (A-CPR) compared to that with MCCD and AED (conventional CPR: C-CPR). Methods: Pigs were randomized into A-CPR or C-CPR groups: The A-CPR group received CPR+ACDA, and the C-CPR group received CPR+MCCD+AED. Hemodynamic parameters, outcomes, and time variables were measured. During a simulation study, healthcare providers performed a basic life support scenario for manikins with an ACDA, MCCD, and AED, and time variables and chest compression parameters were measured. Results: The animals showed no significant in hemodynamic effects, including aortic pressures, coronary perfusion pressure, carotid blood flow, and end-tidal CO_2_, and resuscitation outcomes between the two groups. In both animal and simulation studies, the time to defibrillation, time to chest compression, and hands-off time were significantly shorter in the A-CPR group than those in the C-CPR group. Conclusions: CPR using ACDA showed similar hemodynamic effects and resuscitation outcomes as CPR using AED and MCCD separately, with the advantages of a reduction in the time to compression, time to defibrillation, and hands-off time.

## 1. Introduction

Early cardiopulmonary resuscitation (CPR) and defibrillation are important for successful resuscitation [1,2]. An increase in the rate of bystander and early CPR by bystanders before emergency medical service arrival is associated with an increase in the survival rate after out-of-hospital cardiac arrest [3,4]. However, untrained lay rescuers, even with assistance, may not maintain a high quality of CPR and may not be familiar with using an automated external defibrillator (AED) in cardiac arrest patients [5,6]. Moreover, even if used by healthcare providers, an AED may interfere with chest compressions [7]. As mechanical chest compression devices (MCCD) can provide chest compression of consistent quality, their use has been increasing recently. During the coronavirus disease (COVID-19) pandemic, rescuers have had to take great care to avoid infection by droplet contact given that aerosol exposure events may occur from patients to clinicians, or vice versa, during CPR [8,9,10,11]. The COVID-19 outbreak has created important changes in resuscitation efforts and requires modifications to conventional practices [12,13]. The use of MCCD has increased since the outbreak of COVID-19, and this trend has continued even in the post-pandemic period [14,15].

In many emergency medical services, rescuers bring both MCCDs and AEDs to the scene to rescue victims of cardiac arrest, which can be a physical burden for rescuers. In cases where MCCD and AED are used separately, the rescuer must bring two pieces of medical equipment and operate each device for chest compression and defibrillation separately. Additionally, chest compressions may be interrupted to deliver defibrillation [7]. The development of a device that can perform chest compressions and defibrillation according to a single algorithm by combining an MCCD and AED can solve the problems that arise when using the two devices separately. Therefore, we developed an automated compression–defibrillation apparatus (ACDA) that performs both mechanical chest compression and automated defibrillation. In this study, we investigated the performance of cardiopulmonary resuscitation (CPR) with an ACDA (A-CPR) compared to that with an MCCD and an AED (conventional CPR: C-CPR) in an animal experimental study and a simulation study using manikins.

## 2. Materials and Methods

### 2.1. Automated Compression–Defibrillation Apparatus

**Figure 1:**
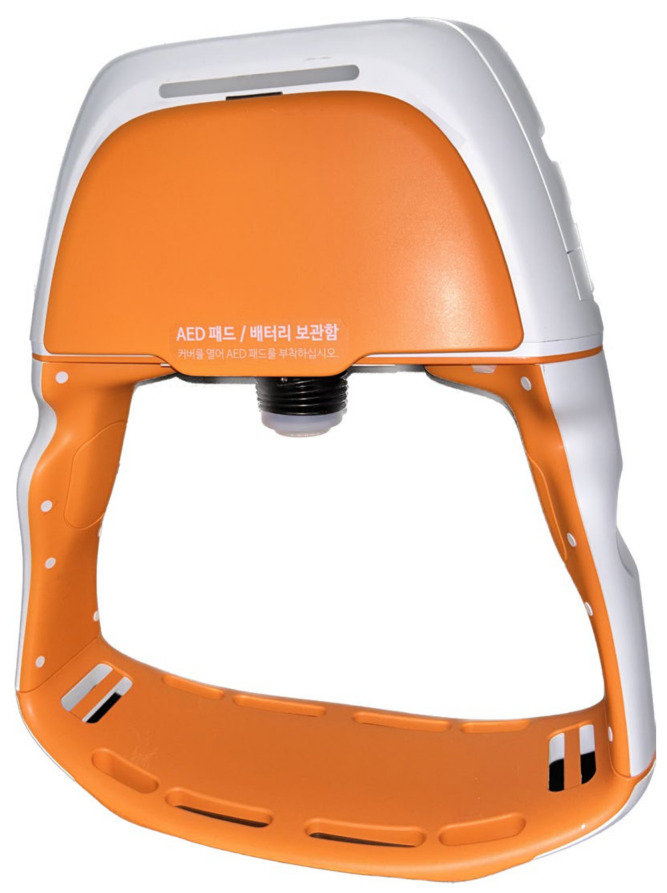
Automatic compression–defibrillation apparatus. The meaning of the Korean notation marked on the equipment is as follows. AED pads and battery storage. Open the cover and take out the AED pads.

The ACDA consisted of a functional unit, supporting structure, and control panel. The functional unit at the top of the apparatus included an AED and an electric motor-powered chest compression device, the supporting structure connected the functional unit to the backboard, and the control panel contained buttons for controlling defibrillation and chest compressions (Figure 1). The chest compression device operates at adjustable compression depths ranging from 0–6 cm and compression rates ranging from 0 to 120 compressions per min. The AED is embedded in a functional unit. With the patient lying on the backrest, the rescuer connects the functional unit to the backboard and attaches the electrode to the patient’s chest, following which the ACDA is ready to operate. During CPR, chest compressions and defibrillation were performed using an algorithm developed according to the CPR guidelines [16]. The ACDA algorithm was configured such that the AED function was performed first, followed by chest compression. Once the ACDA is initiated, it first analyzes the rhythm. If a shockable rhythm is detected, the ACDA starts chest compression and recommends performing defibrillation by pressing the shock button while continuing chest compression. If a non-shockable rhythm is detected, the ACDA immediately performs mechanical chest compressions. If a perfusing rhythm is detected, the ACDA gives voice prompts to check the pulse [17].

### 2.2. Study Design

Both animal experiments and simulations were conducted. An animal experiment using a swine model of cardiac arrest was performed to compare the efficacy, including the hemodynamic effects, of CPR+ACDA or CPR+MCCD+AED. A simulation study using manikins was performed to compare the feasibility of healthcare providers performing CPR+ACDA and CPR+MCCD+AED.

### 2.3. Animal Experiments

#### 2.3.1. Animal Preparation

Animals (Yorkshire pigs weighing 35–45 kg) were fasted overnight for experimental preparation. The pigs had free access to water and food for 12 h before experimentation. Anesthetic induction was initially performed by administering intramuscular ketamine (15 mg/kg) and xylazine (2 mg/kg), followed by inhalation of 3% isoflurane. After endotracheal intubation, the animals were ventilated with a ventilator (Drager Fabius GS, Drager Medical Inc., Telford, PA, USA), with a tidal volume of 10 mL/kg, a positive end-expiratory pressure of 3–5 cm H_2_O, and a respiratory rate of 18 breaths per min. End-tidal carbon dioxide (ETCO_2_) and electrocardiography with lead II were simultaneously monitored during surgical preparation. Aortic pressure was continuously recorded using a 5-Fr micromanometer-tipped catheter (Millar Instruments, Inc., Houston, TX, USA) inserted into the femoral artery. The right atrial pressure (RAP) was recorded using a 5-Fr micromanometer-tipped catheter inserted through the right external jugular vein. A vascular flowmeter (Transonic Systems, Inc., Ithaca, NY, USA) was placed in the right internal carotid artery, and a pacing catheter (5 Fr, bipolar lead; Arrow International Inc., Reading, PA, USA) was introduced via the right internal jugular vein to induce ventricular fibrillation. A total of 2000 units of intravenous (IV) heparin boluses were administered to prevent thrombosis. Baseline arterial blood gas analyses were performed using a blood gas analyzer (i-STAT1, Abbott Laboratories, Abbott Park, IL, USA).

#### 2.3.2. Randomization and Experimental Protocol

After preparation, the animals were randomly divided into A-CPR and C-CPR groups by opening a sealed envelope. The A-CPR group received CPR+ACDA, while the C-CPR group received CPR+MCCD (LUCAS3, Stryker Medical, Kalamazoo, MI, USA) and an AED (AED Plus^®^, Zoll Medical, Chelmsford, MA, USA). The ACDA and MCCD were placed prior to the start of the experiment to prevent catheter dislodgement during CPR. After stabilizing the animal, the baseline hemodynamic data were measured before inducing VF. VF was induced by delivering an alternating electrical current at 60 Hz to the right ventricular endocardium. After 2 min of VF, 6 min of basic life support (BLS) was performed with a 30:2 compression–ventilation ratio. The compression depth was set at 5 cm for ACDA and pre-set at 5.5 cm for MCCD. The compression rate was set to 100/min. Rescue breaths were delivered at a tidal volume of approximately 300 mL using a resuscitator bag (Silicone resuscitator 87005133, Laerdal Medical, Stavanger, Norway). At the end of the 6 min BLS, the AED function of the ACDA in the A-CPR group or the AED in the C-CPR group was turned on. Automated defibrillation sequences were performed according to advanced life support (ALS) guidelines by attaching the electrodes, analyzing the rhythm, and delivering shocks according to voice prompts from the devices [16]. During ALS, continuous chest compressions and intermittent ventilation were administered every 6 s. CPR was performed 20 min after VF induction or ROSC. For ROSC, the animals were euthanized by an IV injection of potassium after 2 h of observation to determine their short-term survival (Supplementary Figure S1).

#### 2.3.3. Measurements and Outcomes

The collected data were digitized using a digital recording system (PowerLab; AD Instruments, Colorado Springs, CO, USA). Aortic and right atrial pressures, end-tidal CO_2_ (ETCO_2_), and carotid blood flow (CBF) were continuously measured and recorded during the experiment. The coronary perfusion pressure (CPP) was calculated in the end-diastolic phase as the difference between aortic and right atrial pressures. The CCF and time variables, including the time to start rhythm analysis, time to shock advice, time to first defibrillation, charge-to-shock interval, hands-off time, and total CPR time, were measured. The outcome measures included successful defibrillation, ROSC, and 2-h survival.

### 2.4. Simulation Study

#### 2.4.1. Participants and Simulation Scenario 

A simulation was conducted using a crossover design. Participants performed CPR using ACDA and CPR using MCCD and AED on a CPR manikin (Laerdal Resusci Anne^®^, Laerdal Medical, Stavanger, Norway) at 1-day intervals. Emergency medical technicians or nurses with BLS qualifications who voluntarily agreed to participate were included in the simulation study. To randomize which simulation to start first, participants selected and opened a sealed envelope containing the simulation type of the first day. The simulation protocol consisted of a scenario in which CPR was performed using an ACDA or MCCD and an AED under the assumption that cardiac arrest was confirmed. Before participating in this study, participants were instructed to watch a video explaining how to use each device and the study protocol. The CPR simulation was started by installing the ACDA or MCCD and finished when the voice prompted a second shock delivery from the ACDA or AED (Supplementary Figure S2).

#### 2.4.2. Measurements

All of the participants’ performances were recorded on video. The video was reviewed by the investigators to calculate the time variables, including the time interval from power-on to the first chest compression, the time from device installation to shock delivery, and the total CPR simulation time. The quality of CPR, including the chest compression depth, rate, and CCF, was recorded and extracted using real-time feedback software (Q-CPR^®^, Laerdal Medical, Stavanger, Norway).

### 2.5. Sample Size

#### 2.5.1. Animal Experiment

On the basis of a previous study reporting a 44 mmHg difference of SBP with a standard deviation (SD) of 35 mmHg between the standard CPR group and alternative CPR group, it was calculated at least 10 subjects would be needed in both groups to provide a statistical power of 80% with a two-sided alpha value of 0.05 [18]. Twenty-four animals were chosen, considering that 20% of animals would be excluded from the analysis due to unpredictable experimental failure.

#### 2.5.2. Simulation Study

On the basis of our ACDA development pilot study, which reported a 20.9-s difference (29.1 ± 7.2 vs. 50.0 ± 12.3) in time to first defibrillation between the A-CPR group and the C-CPR group, the sample size was calculated under the assumption that the time to first defibrillation is reduced by 25% [17]. At least 16 subjects would be needed in both groups to provide a statistical power of 80% with a two-sided alpha value of 0.05. 20 subjects were recruited, considering that 20% of subjects may be excluded from the analysis due to unpredictable events during simulation.

### 2.6. Statistical Analysis

Continuous variables are described as the medians and interquartile ranges and were compared using Student’s *t*-test or the Mann–Whitney U test, as appropriate. Nominal variables were calculated as percentages of the frequency of occurrence and compared using the chi-square test or Fisher’s exact test, as appropriate. In the simulation-based study, the time variables were compared using the paired *t*-test and Wilcoxon rank-sum test. A linear mixed-model analysis was used to compare hemodynamic parameters, including aortic pressure and RAP during the compression and relaxation phases, CBF, CPP, and ETCO_2_, between the two groups. Statistical results are presented as group-time interactions. A *p*-value < 0.05 was considered to indicate statistical significance. Analyses were performed using SPSS software for Windows (version 25.0; SPSS Inc., Chicago, IL, USA).

### 2.7. Statement of Ethical Approval

The animal experiments were approved by the Institutional Animal Care and Use Committee of Yonsei University Wonju College of Medicine, Wonju, Republic of Korea (approval number: YWC-210517-1). The simulation study was approved by the Institutional Review Board of Yonsei University Wonju Severance Christian Hospital (approval number: CR322339).

## 3. Results

### 3.1. Results of the Animal Experiments

Twelve pigs from each group were used in the experiments, with no significant differences in baseline characteristics between the groups except for mean right atrial pressure (Table 1).

#### 3.1.1. Comparison of Hemodynamic Effects and Quality of CPR

The hemodynamic effects, including aortic and right atrial pressures, CBF, CPP, and ETCO_2_, showed no significant differences between the two groups. A difference in right atrial pressure during compression was observed between the two groups, which might be due to the difference in the compression depth settings of ACDA (5.0 cm) and MCCD (5.5 cm) (Table 2, Supplementary Figure S3). The rates of return of spontaneous circulation (ROSC) and 2 h survival did not differ significantly between the two groups (91.7% in the A-CPR group and 100% in the C-CPR group; *p* = 0.262), nor did chest compression fraction (CCF) (93.4% [90.4–97.4] in the A-CPR group, 91.5% [87.2–96.8] in the C-CPR group, *p* = 0.322) (Table 3).

#### 3.1.2. Comparison of Time Variables

**Figure 2:**
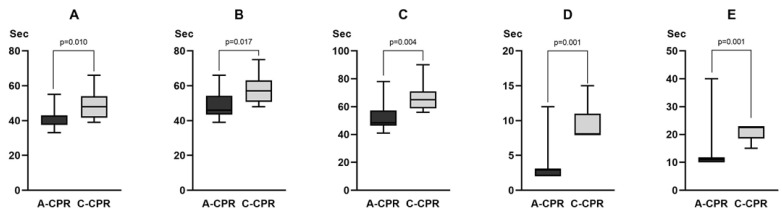
Comparison of the time variables between two groups in the animal experiment. (**A**) Time for rhythm analysis. (**B**) Time to shock advice. (**C**) Time to first defibrillation. (**D**) Charge-to-shock interval. (**E**) Hands-off time. For the value of each time variable, refer to the main text.

The time to rhythm analysis (38.0 s [37.5–43.0] vs. 48.0 s [41.8–54.0], *p* = 0.010), time to shock advice (46.0 s [43.5–54.3] vs. 57.0 s [50.8–63.0], *p* = 0.017), time to first defibrillation (48.5 s [46.3–57.3] vs. 65.0 s [58.8–71.0], *p* = 0.004), charge-to-shock interval (3.0 s [2.0–3.0] vs. 8.0 s [8.0–11.0], *p* = 0.001), and hands-off time (11.0 s [10.0–11.8] vs. 22.5 s [18.5–23.0], *p* = 0.001) were significantly shorter in the A-CPR group than those in the C-CPR group (Figure 2). 

### 3.2. Results of the Simulation Study

**Figure 3:**
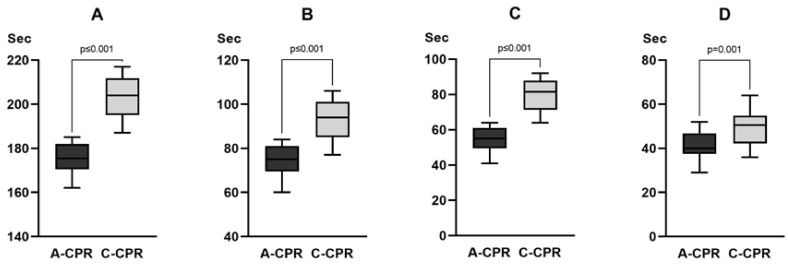
Comparison of the time variables between the two groups in the simulation study. (**A**) Total CPR simulation time. (**B**) Hands-off time. (**C**) The time interval from power-on to first chest compression. (**D**) Time to the first shock delivery. CPR = cardiopulmonary resuscitation. For the value of each time variable, refer to the main text.

Eighteen emergency medical technicians and two nurses participated in the simulation study. The total CPR simulation time was significantly shorter in the A-CPR group than that in the C-CPR group (176 s [171–182] vs. 204 s [195–212], *p* < 0.001). The hands-off time (75 s [70–81] vs. 94 s [85–100], *p* < 0.001), the time interval from power-on to first chest compression (55 s [50–61] vs. 82 s [72–87], *p* ≤ 0.001), and the time to the first shock delivery (40 s [38–47] vs. 51 s [43–55], *p* = 0.001) were significantly shorter in the A-CPR group than those in the C-CPR group (Figure 3). The CCF was significantly higher in the A-CPR group than in the C-CPR group (57% [56–59] vs. 54% [53–56], *p* < 0.001) (Figure 3). The chest compression rate was significantly higher in the C-CPR group (5.0 cm [4.9–5.1] vs. 5.6 cm [5.5–5.7], *p* ≤ 0.001), and the chest compression rate was significantly higher in C-CPR than in A-CPR (100 [100–100] vs. 102 [102–102], *p* < 0.001). Additionally, we observed a significant difference in the rate of accurately performing chest compressions with a depth of >5 cm CPR between the two groups (100% [98–100] in A-CPR, 97% [97–97] in C-CPR, *p =* 0.003) (Supplementary Table S1).

## 4. Discussion 

Our study showed that CPR using ACDA was similar to CPR using MCCD and AED in terms of hemodynamic effects and resuscitation outcomes in animal experiments. In a manikin simulation study, regarding the quality of CPR, CPR using ACDA was similar to that using MCCD and AED. However, both animal experiments and simulation studies showed that CPR using ACDA shortened the time required for each stage of resuscitation and hands-off time compared to CPR using MCCD and AED. These results indicate that ACDA, which combines the MCCD and AED into one device and operates with one operating algorithm, is more time-efficient than using the MCCD and AED separately. To the best of our knowledge, the ACDA is the first medical device to integrate an MCCD and an AED operated by a unified algorithm that controls chest compressions and automatic defibrillation. Our study demonstrates that the integration of these two essential pieces of equipment for CPR may contribute to increased survival by shortening the time required for resuscitation.

Chest compressions are frequently interrupted during resuscitation due to assessment and interventions such as analysis of cardiac rhythm, delivery of shocks, airway management, vascular access, and drug administration [19]. It has been shown that during basic life support and AED use by first responders, CPR is not performed during 23% of the CPR time due to programmed interruptions, such as AED connection time [20]. Interruption of chest compressions may adversely affect hemodynamic effects and resuscitation outcomes. Moreover, in an animal study, CPP and left ventricular blood flow were found to decrease during the interruption of chest compressions for rescue breathing [21]. In patients with ventricular tachycardia/fibrillation in out-of-hospital cardiac arrest, a 5-s increase in the longest overall pause was associated with lower odds of survival [22]. In our animal experiments and simulations, the time required for automated defibrillation and hands-off was shorter in the A-CPR group than in the C-CPR group. A-CPR can reduce the time to defibrillation and hands-off time per CPR cycle by approximately 11 s each compared to C-CPR; therefore, if CPR cycles are repeated during prolonged CPR, a cumulative shortening effect may occur. ACDA controls automatic defibrillation and chest compressions using a single algorithm and performs defibrillation without stopping the chest compressions, thereby shortening the time until defibrillation and hands-off time.

Manual CPR has been challenged by factors related to the risk of infection transmission or mental/physical stress on rescuers [10,23]. MCCD represents a practical alternative to manual CPR during pandemic periods or in cases where manual CPR cannot be performed. Together with AEDs, MCCDs have become essential equipment rescuers carry at the scene of cardiac arrest, particularly during the COVID-19 pandemic when the risk of infection was a particular concern. Carrying the AED and MCCD to the scene and operating the two devices separately can burden rescuers. Rescuers may also perform additional efficient resuscitation techniques such as extracorporeal CPR or vector change defibrillation, as the use of this device reduces the burden on the rescuer and enables continuous resuscitation. The integration of chest compression and defibrillation within a device makes the seamless implementation of resuscitation possible. The use of ACDA may be the first step in integrating essential medical devices to make CPR more effective and comfortable. 

Our study has some limitations that warrant discussion. First, as animal experiments were performed to evaluate hemodynamic efficacy and resuscitation outcomes, our results are limited in their generalizability to humans. Second, to prevent catheter dislodgement during animal experiments, the basic life support sequence was started after the equipment was applied to the animal in advance; thus, the time intervals measured in the animal experiments did not include the equipment application time. Moreover, the results of the simulation study, which included the equipment application time, time to compression, and time to first defibrillation, were shorter in the A-CPR group than those in the C-CPR group. Because the ACDA used in this study was developed as a prototype, there is room for further development in the future. Although there was no statistical significance, the 8.3% difference in ROSC rate in favor of C-CPR needs to be confirmed through additional studies.

## 5. Conclusions 

CPR using ACDA, which combines AED and MCCD, shows similar hemodynamic effects and resuscitation outcomes as CPR using AED and MCCD separately. Moreover, CPR using ACDA has time-saving effects, including a reduction in the time to compression, the time to defibrillation, and hands-off time.

## Figures and Tables

**Table 1:** Comparison of baseline characteristics in the animal experiment.

Characteristics	A-CPR (n = 12)	C-CPR (n = 12)	*p*-Value
Male, n (%)	10 (83.3)	7 (58.3)	0.178
Body weight (kg)	36.5 (36–37.75)	38.0 (36.25–39.50)	0.167
Systolic blood pressure (mmHg)	105 (94–118)	97 (92–106)	0.128
Diastolic blood pressure (mmHg)	69 (60–78)	67 (57–72)	0.185
Mean aortic pressure (mmHg)	82 (70–92)	77 (68–83)	0.154
Mean right atrial pressure (mmHg)	2.2 (1.0–3.5)	4.2 (3.1–5.4)	0.039
CPP (mmHg)	86 (68–95)	80 (60–86)	0.396
CBF (mL/min)	206 (165–264)	186 (149–232)	0.382
ETCO_2_ (mmHg)	49 (46–53)	49 (44–50)	0.248
pH	7.473 (7.439–7.498)	7.493 (7.462–7.533)	0.141
PaCO_2_ (mmHg)	35 (28–41)	31 (29–37)	0.488
PaO_2_ (mmHg)	111 (100–134)	118 (96–141)	0.954
Bicarbonate (mmol/L)	27.8 (20.2–29.4)	25.4 (22.2–29.9)	0.885
SaO_2_ (%)	99 (98–99)	99 (98–99)	0.570
Lactate (mg/dl)	1.3 (1.0–2.5)	0.3 (0.7–1.5)	0.106

Variables are presented as the median (interquartile range) or frequency (percent). CPR = cardiopulmonary resuscitation; A-CPR = CPR with automatic compression–defibrillation apparatus; C-CPR = CPR with mechanical chest compression device and automated external defibrillator; CPP = coronary perfusion pressure; CBF = carotid blood flow; ETCO2 = end-tidal carbon dioxide; PaCO_2_ = arterial carbon dioxide pressure; PaO_2_ = arterial oxygen pressure; SaO_2_ = arterial oxygen saturation.

**Table 2:** Comparison of hemodynamic effects during CPR between two groups in the animal experiment.

Hemodynamic Parameters	Elapsed Time after CPR	2 min	4 min	8 min	12 min	16 min	20 min	*p*-Value
Aortic pressure, compression (mmHg)	A-CPR	94 (89–101)	92 (14–105)	91 (68–103)	131 (85–161)	102 (78–133)	71 (35–106)	0.186
	C-CPR	86 (81–105)	96 (76–102)	99 (90–107)	107 (105–122)	166 (104–276)	104 (75–134)	
Aortic pressure, relaxation (mmHg)	A-CPR	27 (22–32)	18 (15–22)	18 (13–22)	35 (32–41)	30 (17–33)	24 (3–45)	0.256
	C-CPR	14 (11–21)	16 (9–21)	20 (13–26)	34 (16–42)	64 (26–126)	26 (24–28)	
Mean aortic pressure (mmHg)	A-CPR	47 (46–50)	42 (39–48)	42 (39–48)	67 (56–75)	54 (38–66)	39 (14–65)	0.168
	C-CPR	52 (36–52)	45 (37–49)	46 (41–50)	59 (48–65)	98 (52–176)	52 (41–63)	
Right atrial pressure, compression (mmHg)	A-CPR	113 (76–149)	155 (55–254)	159 (119–198)	131 (95–167)	113 (77–149)	121 (87–155)	0.023
	C-CPR	160 (115–206)	139 (114–163)	128 (119–138)	106 (91–121)	90 (73–106)	65 (24–105)	
Right atrial pressure, relaxation (mmHg)	A-CPR	4 (3–4)	5 (5–6)	6 (5–7)	6 (6–6)	8 (8–8)	7 (6–7)	0.099
	C-CPR	4 (3–5)	3 (3–3)	3 (2–4)	5 (4–5)	5 (3–6)	4 (2–5)	
CPP (mmHg)	A-CPR	43 (36–49)	35 (26–47)	34 (28–45)	45 (36–78)	45 (32–55)	34 (11–56)	0.385
	C-CPR	13 (5–17)	14 (6–19)	17 (5–22)	35 (21–39)	72 (21–132)	48 (37–59)	
CBF (mL/min)	A-CPR	298 (226–360)	318 (240–390)	293 (230–351)	215 (175–266)	252 (185–292)	253 (113–393)	0.768
	C-CPR	361 (258–407)	357 (329–434)	381 (295–398)	194 (113–239)	149 (94–247)	78 (65–90)	
ETCO_2_ (mmHg)	A-CPR	30 (26–31)	20 (17–29)	25 (23–34)	34 (30–44)	33 (30–35)	33 (24–41)	0.591
	C-CPR	24 (22–28)	21 (18–27)	25 (22–41)	28 (24–37)	37 (28–47)	26 (24–29)	

Variables are presented as the median (interquartile range). CPR = Cardiopulmonary resuscitation; A-CPR = CPR with automatic compression–defibrillation apparatus; C-CPR = CPR with mechanical chest compression device and automated external defibrillator; CPP = coronary perfusion pressure; CBF = carotid blood flow; ETCO_2_ = end-tidal carbon dioxide.

**Table 3:** Comparison of outcomes between the A-CPR and C-CPR groups in the animal experiment.

Outcomes	A-CPR (n = 12)	C-CPR (n = 12)	*p*-Value
Total number of defibrillations	2.5 (1.0–5.3)	3 (1.0–4.8)	0.893
CCF (%)	93.4 (90.4–97.4)	91.5 (87.2–96.8)	0.322
ROSC	11 (91.7%)	12 (100%)	0.262
2 h survival	11 (91.7%)	12 (100%)	0.262

Variables are presented as the median (interquartile range) or frequency (percent). CPR = cardiopulmonary resuscitation; A-CPR = CPR with automatic compression–defibrillation apparatus; C-CPR = CPR with a mechanical chest compression device and automated external defibrillator; ROSC = return of spontaneous circulation; CCF = chest compression fraction.

## Data Availability

The data used to support the findings of this study are available from the corresponding author upon request.

## Supplementary Materials

The following supporting information can be downloaded at: https://www.mdpi.com/article/10.3390/jcm12165333/s1, Figure S1: Automatic compression-defibrillation apparatus applied to an animal for experiments; Figure S2: Animal experimental protocol; Figure S3: Simulation scenarios. Figure S4: Comparison of hemodynamic effects during CPR between two groups in the animal experiment; Table S1: Comparison of the quality of CPR between the A-CPR and C-CPR groups in the simulation study.
