# Supplementary material for: Efficacy of Cardiopulmonary Resuscitation Using Automatic Compression—Defibrillation Apparatus: An Animal Study and A Manikin-Based Simulation Study
Source: J Clin Med. 2023 Aug 16;12(16):5333. doi: 10.3390/jcm12165333 (PMC10455516; doi:10.3390/jcm12165333)
Supplement: Supplementary file 1 [file jcm-12-05333-s001.zip › jcm-2544405-supplementary.pdf]

Supplementary Figure S1. Automatic compression-defibrillation apparatus applied to an animal for experiments.

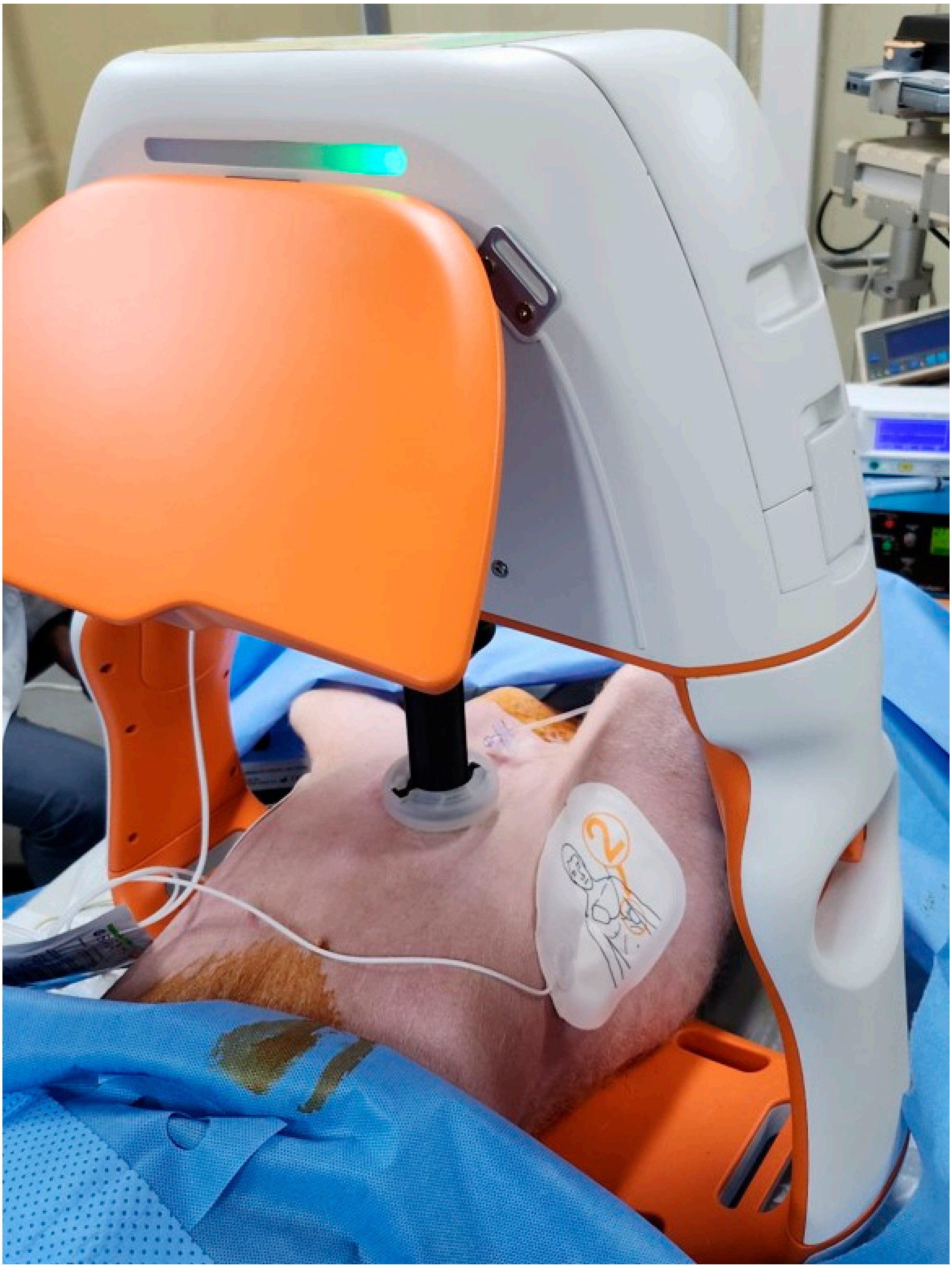

Supplementary Figure S2. Animal experimental protocol.

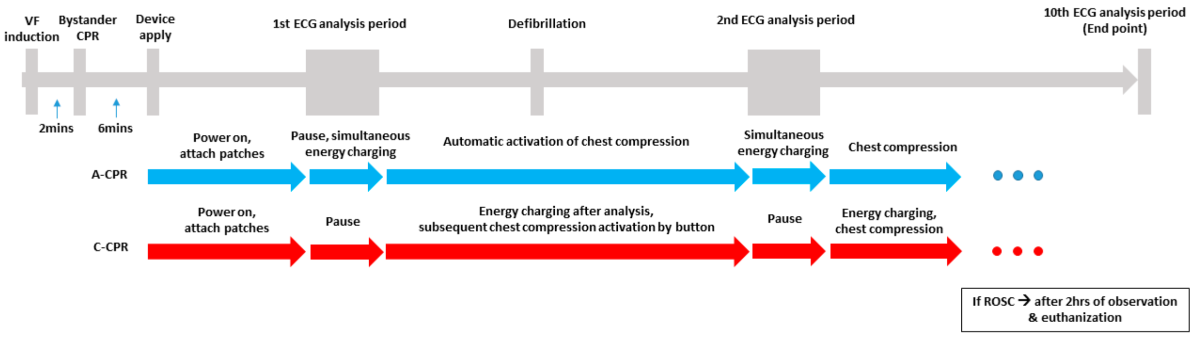

VF: Ventricular fibrillation, A-CPR: CPR with automatic compression-defibrillation apparatus, C-CPR: CPR with a mechanical chest compression device and automated external defibrillator, ROSC: Return of spontaneous circulation.

Supplementary Figure S3. Simulation scenarios.

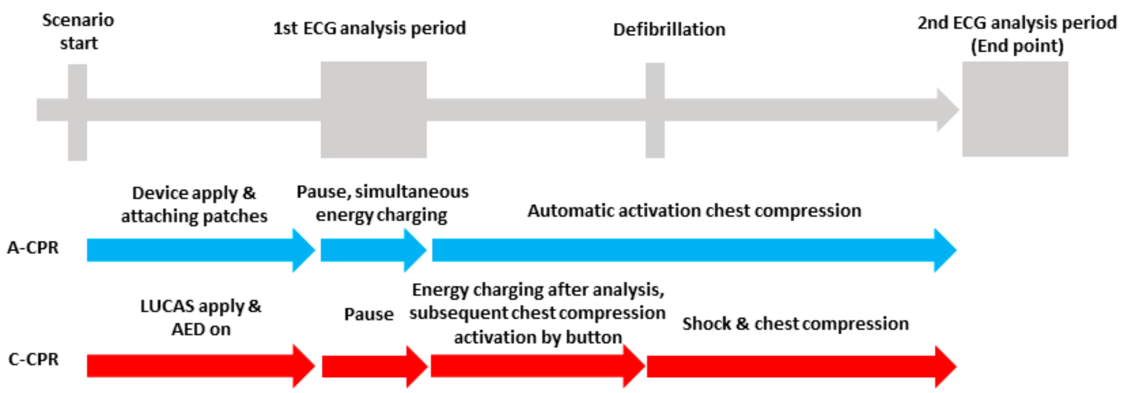

A-CPR: CPR with automatic compression-defibrillation apparatus, C-CPR: CPR with a mechanical chest compression device and automated external defibrillator.

Supplementary Figure S4. Comparison of hemodynamic effects during CPR between two groups in the animal experiment. (A) Aortic pressure, compression phase (B) Aortic pressure, relaxation phase. (C) Mean aortic pressure (D) Right atrial pressure, compression phase (E) Right atrial pressure, relaxation phase (F) Coronary perfusion pressure (G) Carotid blood flow (H) End-tidal carbon dioxide. For the value of each time variable, refer to the main text.

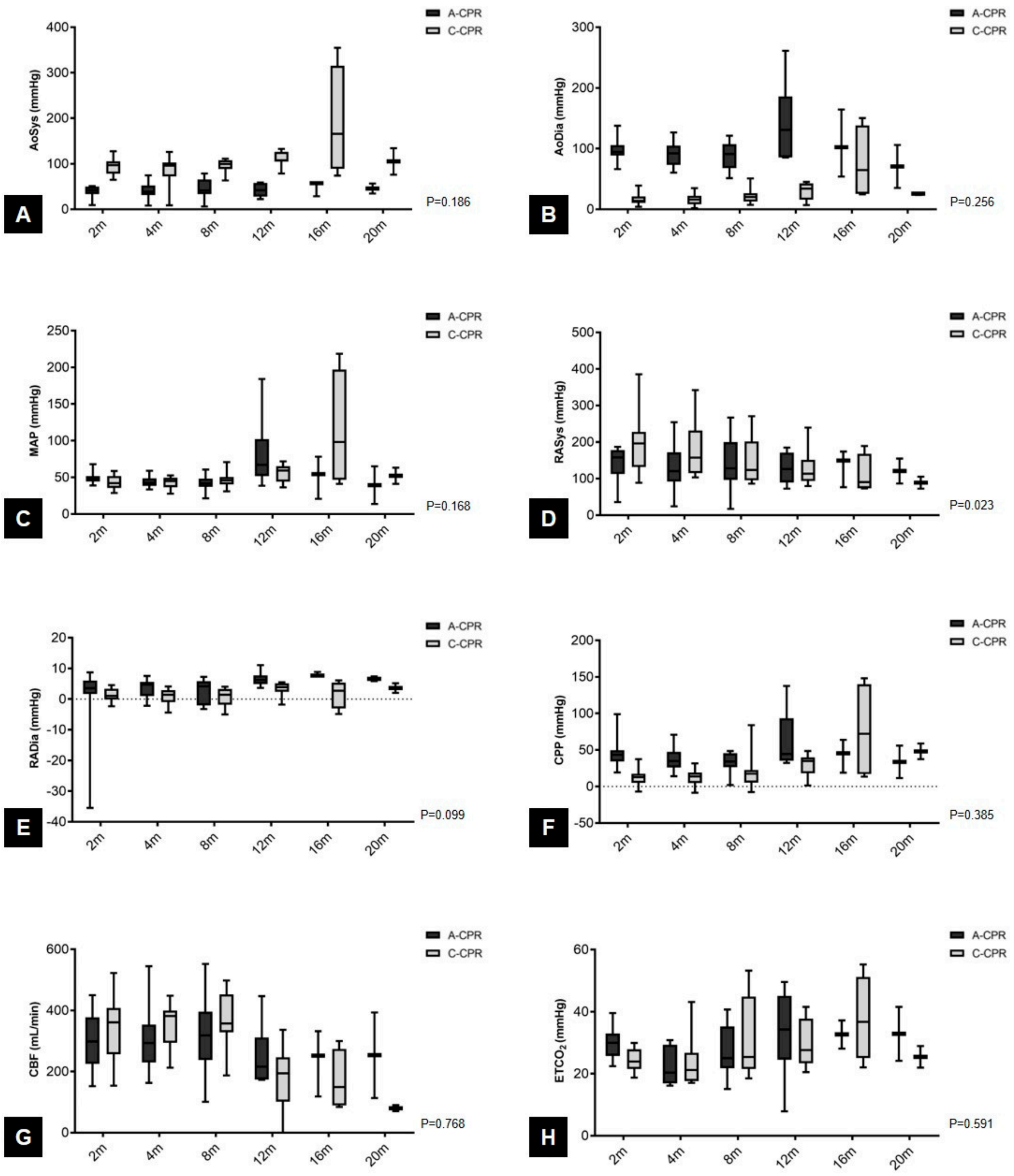

Supplementary Table S1. Comparison of the quality of CPR between the A-CPR and C-CPR groups in the simulation study

Chest compression parameters	**A-CPR (n = 20)**	**C-CPR (n = 20)**	***p*-value**
Compression fraction (%)	57 (56–59)	54 (53–56)	< 0.001
Compression depth (cm)	5.0 (4.9–5.1)	5.6 (5.5–5.7)	< 0.001
Compression rate (/min)	100 (100–100)	102 (102–102)	< 0.001
Optimal compression depth (≥ 5 cm, %)	100 (98–100)	97 (97–97)	0.003

Variables are presented as median (interquartile range) or frequency (percent)

CPR: Cardiopulmonary resuscitation, A-CPR: CPR with automatic compression-defibrillation apparatus, C-CPR: CPR with mechanical chest compression device
